# Emotions as Key to Russian GenZs’ Consumption of Political News

**DOI:** 10.11621/pir.2022.0203

**Published:** 2022-06-15

**Authors:** Elena A. Salikhova, Daria M. Vyugina

**Affiliations:** a Lomonosov Moscow State University, Moscow, Russia

**Keywords:** Digital generation, generation Z, media consumption, political agenda, political news, emotions

## Abstract

**Background:**

Media consumption by the digital generation in Russia should not only be of interest to media researchers or managers. The fact that young people’s interaction with various information sources and the media is noticeably different from the daily practice of older generations and has created new trends and habits, must be taken into account by the teaching community, parents, and political forces. This issue is of particular interest to politicians, because it points to the need for them to transform their information policy.

**Objective:**

The purpose of this study was to find out how media formats that appeal to emotions of young people influence their media consumption.

**Design:**

During the first stage, we surveyed respondents in three cities with populations of over one million; the surveys allowed us to identify key sources of information and the motivation for various kinds of digital content consumption by youth. During the second stage, 20 in-depth interviews provided a deeper understanding of where the teenagers got their political news and what influenced their consumption.

**Results:**

The authors of this paper have concluded that the digital generation does not consume political news purposefully. They rarely turn to socio-political publications and do not watch shows on federal TV channels. In fact, members of this generation mostly deny having any interest in this topic or awareness of it. However, through various entertaining, primarily humorous, content, which is the most popular among young people, a clear political agenda, albeit subjective, is being formed in their information space.

**Conclusion:**

Our work refutes the common misconception of researchers, representatives of the state system, and journalists that the digital generation is not interested in politics. Rather, their pursuit of emotional experiences, primarily positive ones, has become their main incentive for consuming political news.

## Introduction

The media consumption of the digital generation in Russia should not be only of interest to media researchers or media managers. The fact that the young people’s interaction with various information sources and the media is noticeably different from the daily practice of older generations and has created new trends and habits, must be taken into account by the teaching community, as well as parents and political forces.

Historically determined features of Russia’s political system, its stagnation in some areas, and the fact that it relies on the older generation’s interests have led the government to rely on federal television in the hope that it will be the key, if not the only, source of political information for the population. At the same time, alternative information channels have been actively developed, such as Internet media, social networks, and messenger channels, which are most actively used by Russian youth searching for diverse content on the Internet.

For this reason, youth are less susceptible to state propaganda and have many different sources of information (both reliable and not) than representatives of previous generations. Political forces today do not always understand which channels to choose for interaction with the younger generation. Of course, this does not mean that young people are shielded from propaganda; this is impossible, if only because not all members of the digital generation are able to choose accurate sources of information on the Internet.

At first glance, it appears that political content rarely becomes the focus of attention of Russian young people (Mukhametshina, 2020); they prefer influencer news, entertainment content, and game formats. However, a deeper analysis shows that young audiences’ need for positive emotions indirectly motivates their consumption of political news about events in the country and in the world, and also serves as a window into all key newsworthy information.

The attitude of the digital generation toward political news and topics highlights the contradiction that underlies this generation (Vyugina, 2019). On the one hand, young people are very often accused of apathy, disinterest in the political agenda, and most importantly, unwillingness to take part in the important social processes in the country. In the United Kingdom, for example, young people are blamed for Brexit, since it was young people who did not come to the polls, even though statistics showed that they were against the country’s withdrawal from the European Union. In the United States, youth are accused of mass riots and of lacking a clearly formulated position toward ex-President Donald Trump.

However, critics of today’s youth often forget that every generation passes through a stage where they are isolated from current events and are not interested in politics in general. Such passive behavior is explained by the youth community’s engagement with their own lives, inner world, personal issues, and local conflicts. The digital generation needs time to grow up, discover its identity, and formulate their positions (Scholz & Vyugina, 2019).

At the same time, young people are involved in the nation’s political life through their emotional lives. The nature of emotions and their significance during communication and a person’s social life were discussed in detail in the book *Psychology of Emotions* by Carol Izard.

Izard defines emotion as “one of the hallmarks of humanity” (Izard, 1991), “... something that is experienced as a feeling that motivates, organizes, and directs perception, thoughts, and action” (Izard, 1991). Various aspects of emotions are also considered in the works of R. Collins (2014), P. Ekman (2007), J. Jasper (2014), L.Ya. Gozman (1987), T. Kemper (2011), R. Plutchik (1980), J.H. Turner (2007), T. Sheff (2002), and others. Today the study of the influence of social networks on people’s emotional sphere, and especially that of young people, is an important area of research (Soldatova & Rasskazova, 2017; Soldatova & Teslavskaya, 2018; Voiskunskii & Soldatova, 2019; Turkl, 2010; and others).

One of the most reputable Russian journalists, V. Posner, considers political censorship short-sighted: “Restrictions on media freedom, which we have been witnessing for the last 10-12 years [...], lead to more and more people looking for other sources of information [...]” (Posner, 2017). For young people, memes are becoming one of the alternative sources, which are actively distributed through social networks and messengers.

In addition, researchers have noted that “in the context of the increasing role of social networks and smartphone applications, news games are one of the most needed formats to increase user engagement in media content” (Krasheninnikova & Zatsepilina, 2019). These tools provide the materials with a fresh look, show the event from an unusual perspective, and give each user the opportunity to feel like a participant in the events. Game formats in online media, in addition to having a completely different emotional effect than objective reporting, allow the audience to consider details that usually remain behind the scenes and draw their own conclusions. As a result, it is much more difficult to conceal the truth and impose an unambiguous interpretation of events in such formats. In social networks, even negative events are often broadcasted in the form of memes and funny comments; it becomes easier to accept what happened in this context (Levada Centre, 2020, recognized as a foreign agent by Russian government).

We consider memes and games as important cultural phenomena of our time. Their entertainment and information functions and penetration into the media environment are directly related to the phenomenon of infotainment. Recall that infotainment represents a blurring of the line between news and entertainment. Media researchers see a particular danger in the pressure of infotainment on the political news agenda. Bourdieu noted that the focus is on “those things that may arouse curiosity, but do not require analysis, especially in the political sphere” (Bourdieu, 1999). E. Vartanova emphasized that “[...] informing the audience and events analysis are made as easy as possible, and news programs present politics as a sphere of mass culture.” (2003) However, the growth of entertainment content does not mean a departure from politics; on the contrary, politics simply acquires new forms and becomes more attractive to young media consumers.

The meme phenomenon has emerged relatively recently, but due to its prevalence and significant role in modern network communication, a fairly broad academic interest in this phenomenon can be observed (Artyomova, 2002; Budovskaya, 2013; Yershov, 2019; Kanashina, 2017; Krongauz, 2014; Savitskaya, 2013; Shomova, 2019; etc.). The Oxford English Dictionary defines a meme as “an element of culture that is transmitted through non-genetic channels, that is, through imitation” (Budovskaya, 2013). Media researchers, cultural scientists, political scientists, and sociologists also note the connection of a meme with the cultural environment and genetic cultural memory, and consider it as a tool for transmitting a nation’s cultural code. The author of the learning guide “Memes as they are,” S.A. Shomova, provides the following definition of an Internet meme: “It is a specific type of messages on the Network, combining short statements of various genres and diverse in semiotic nature, on topics relevant to the network community and having at the same time a viral nature — thanks to the brightness and semantic content, flamboyance and/or visual ‘packaging’ ” (2019).

We consider the meme to be a creolized text. A creolized text is understood as a text that uses both verbal (textual) and nonverbal (visual, graphic) channels of information transmission. The phenomenon of a creolized text is investigated in the works of Lotman (1999), Plotnikov (1992), and Eco (1998). Since visual images are given a key place in the meme’s semiotic system, this determines their popularity among the youth audience.

The semiotic system of the meme also includes ideological, cultural, and social codes. A ‘code’ is a system of signs/symbols that are understandable and shared by the majority of participants in the cultural space. Decoding a meme involves identifying the complex relationships in a system of codes. The need to decode a meme is another reason for the huge popularity of this content format. While a text or article arranges everything in orderly pigeonholes, a meme involves overcoming an obstacle, a quest, an exciting cognitive process that brings satisfaction when its meaning is successfully deciphered. “I like it when I understand why everyone is laughing,” respondents said in an interview. They confessed: “When I do not know what the meme is about, I feel uncomfortable, I go quickly and google it.” This illustrates the fact that decoding also performs the “friend-foe” function.

The extreme popularity of the meme is also explained by its humorous nature and the aptitude of the Internet audience for humor (Shomova, 2019). In our qualitative interviews, respondents noted that their main ways to relax during the intervals between intense training sessions were short funny videos and memes found on the Web, which young people constantly send to each other in order to cheer up and distract themselves.

A meme is focused on entertainment. The political memes we used in this study were responses to socially significant events taking place in the country and in the world. They were not only entertaining, but also had satirical connotations. The satire of the meme involved ridiculing and criticizing news occurrences. Both the entertaining and satirical orientation are explored in the paradigm of humor in culture, which is distinguished by the critical reversal of values (Bakhtin, 1999). Memes include features of a newspaper satire (language code) and political caricature (language and visual codes) at the level of genre features (Kanashina, 2017). Both newspaper satires and caricatures are characterized by journalistic saturation; reality is symbolically interpreted, which leads to the creation of satirical images.

Another important aspect of the meme is its immediacy, and as a result, short lifespan. The image (drawing, photo, or collage) that underlies the meme and is accompanied by a certain text always illustrates something temporary, thereby activating consumers’ interest in the current agenda. This peculiarity of the meme perfectly correlates with its effectiveness in transmitting political content: one news item quickly replaces another and is updated, so it is important not only to understand the irony or satire embedded in the illustration, but also to understand the context. Therefore, media researchers pay attention to the effects of memes, especially in the context of political communication: the viral nature of the meme does not just “infect” a person’s mind but is sometimes able to change his behavior and mental attitudes (Shomova, 2019).

Social human activities were “riddled with games” (Huizinga, 1980) from the very beginning. The game phenomenon is an object of interdisciplinary research of philosophers, culturologists, sociologists, and psychologists (Salikhova, 2021). In psychological papers, a game is considered as the most important phenomenon in the development of the child’s personality (Vygotsky, 2004; Zaporozhets, 1986; Piaget, 1994; Leontiev, 1974; Guseynov, 2009; Elkonin, 1999; etc.). In the 1970s computer games arose, and in the 1990s the era of dynamic growth of the gaming industry began. Psychological science began to explore the features of gaming activity in the digital environment; the impact of passion for video games on the physical and psychological health of children and adolescents and their cognitive abilities (Voiskunsky et al., 2017; Rubtsova, 2019; Soldatova & Teslavskaya, 2017); and the motivational approaches to online gaming (Bartle, 2003; Csikszentmihai, 1990; Demetrovic et al., 2001; Desi & Ryan, 1991; Hainey et al., 2011; Ivanova, 2017; Voiskounsky & Wang, 2014).

Researchers believe that good games always satisfy needs: not only situational, but also universal basic needs. This can explain the enormous popularity of computer games around the world (Ivanova, 2017). The exceptional success of computer and video games (we use these words interchangeably) led journalists to think about using gaming techniques in journalism to reach a young audience, which is also called the generation of gamers. As part of our study, we asked the question “What do you do on the Internet most often?” (Multiple answers could be selected). Almost 19% (18.8%) of respondents reported that they play online games. It means almost every fifth student is interested in online games. Among schoolchildren, this number is much higher; 36.2% of respondents play online games on a regular basis.

The terms “games with news” and “news games” will be used further on. By “games with news,” we mean the use of the information agenda in the gaming industry. “News Games” are the games created by journalists who are looking for innovative formats to involve a youth audience in media consumption. It is worth adding that the political aspects of the game as a new format of political communication have been studied relatively recently (Bogost & Ferrari, 2010; Potorochina, 2016; Shomova, 2004).

The advantages of the game format include the news’ impact on the consumer’s emotional sphere, since pleasure and fun are considered as the most important characteristics of the game (Salikhova, 2021). The game is also distinguished by a high level of involvement in the content consumption.

The news game’s potential is not in reporting current news, but in diving into the context and background environment associated with the news (Burton, 2005). Such immersion facilitates the creation of unique content and attracts the audience to major publicly significant social, historical, or political issues (Plewe & Fürsich, 2017; Wolf & Godulla, 2018).

Media companies are much less likely than the gaming industry to turn to game formats when covering the political issues: games require large resources, primarily development time, while it is important for political news to retain its relevance. But let us venture to suggest that the volume of gamified political content will grow. The authors’ assumption is based on the fact that the statistics are similar for all countries: according to VCIOM (VCIOM, 2017), young people have minimal interest in political processes and distance themselves from politics and politicians. News games, on the other hand, make political topics interesting, attractive, and engaging in the eyes of youth. This should be realized by the media, which need to accustom young people to the political world, and by political parties, which need to replenish the ranks of their supporters.

The interactive nature of the formats we’ve mentioned plays an important role in their ability to develop interest, be tolerated, and even create a kind of addiction. Interactivity provides immersion and maximum involvement in the content, the strengthening of an emotional connection between the user and the content. Young people are used to constant interaction with content. Reading media means commenting, striking likes, and sharing content for them. Therefore, one cannot but agree with the researcher Yu.M. Yershov, who notes that “without personal involvement in the media process, teenagers’ interest in watching or reading is rapidly fading” (2019).Note also that there is a fundamental difference between a meme and a game. The meme is anonymous in most cases; it is the product of folk art and a genre of folk humor, which brings it closer to a popular joke. Based upon the memes’ popularity, collections of memes often appear in the media; they can be related to a specific news topic, or identifying the most popular memes of a week, a month, or a year. The game is the product of planning, a format that requires financial investments, and longterm work by various editorial departments: programmers, designers, and journalists (Salikhova, 2020).

Our research showed that when respondents were talking about the content they share with each other daily, they constantly mentioned ironic pictures, cartoons and collages (memes less often), tests, and games, which led to the hypothesis that their main source of socio-political information was new, alternative formats of political news, which spread rapidly in the digital space. First, viral entertainment content is most in demand within the age category we are studying (Google, 2017; Yershov, 2019). Second, obtaining information about socio-political events through new formats is the result of the media regulation and the shunning of alternative political positions by federal TV channels and print media.

## Methods

### Participants

At the first stage of our study as a part of a grant research, we conducted a survey of respondents’ media consumption. The following conditions were used as the basis for selecting the sample: the survey was to cover three cities; the distribution of the sample by city should be uniform; and the sampled population for each of the cities should comprise representatives of generation Z*, i.e.*, the aggregate of children and youth from 10 to 19 years old studying in secondary educational institutions and universities (from the 5th grade of school to the 2nd year of university). On the basis of these conditions, we designed a set of 4320 questionnaires divided into two types on the basis of age of the respondents. In each city, 960 middle school students (1017 years old) were to receive questionnaires, and 480 university students (17-19 years old) were to receive them. Altogether, that amounts to 2,880 middle school students and 1,440 university students.

A codifier was created for each of the open questions, and all the respondents’ answers were translated into digital indicators. A total of 246 codes were created. Then an input layout was created in the SPSS package for processing a common data array. Thus, linear distributions of the responses to each question were obtained. The data was processed in the SPSS package.

Ultimately, 1471 questionnaires were received from universities: 498 (33.9%) from Moscow; 492 (33.4%) from Nizhny Novgorod; and 481 (32.7%) from Rostovon-Don. This was 31 participants more than originally planned.

As for the schools, 990 school questionnaires were received in each of the cities participating in the study, adding up to 2970 in total, which exceeded the original sample design by 90 questionnaires. The collected data array was sufficient to conduct theoretical research that complies with the principles of reliability.

### Procedure

We formulated several hypotheses in the course of our work:

*Hypothesis 1.* The popularity of entertainment content among young people has led to the emergence of new formats for presenting political news, which are actively used by media that is popular among this generation;

*Hypothesis 2.* Generation Z consumes political news mainly in entertaining and often humorous formats, so their understanding of the situation in the world and in Russia is very subjective; and

*Hypothesis 3.* The main motive for the consumption of entertainment content by generation Z is their need for positive emotions, so they treat political news lightly, with humor, and without critical evaluation/comprehension.

The first stage of the study helped to determine the importance of entertainment content as a key source of information for Generation Z.

In the second stage of the study, in order to obtain in-depth data on the specifics of the political content consumption, an additional qualitative study was conducted, namely, in-depth interviews with 20 respondents. This sample cannot be called representative, but it provided the opportunity to assess the particularities of the political agenda developed among the youth. Each of the generation Z members was asked to analyze six newsworthy occurrences related to the internal policy of the Russian Federation, which were presented in a visual humorous (memes and collages) or game format, and then to share the emotions they felt while interacting with these types of content.

The political news was selected according to its scale and importance: the epidemic of coronavirus (and the subsequent regime of self-isolation); the discussion of the constitutional amendments and “zeroing” of Vladimir Putin’s term; and the protest movement in Belarus. These became the key topics of spring and summer 2020, including for the digital generation, according to a study by the Levada center* (*recognized as a foreign agent by Russian government, Goncharov & Karaeva, 2020). This fact indicated that the youth’s news agenda does not differ significantly from the all-Russian agenda.

Respondents were offered about 30 memes, as well as five game projects published in the media during this time period, dedicated to the same political news topics.

## Results

Our study of media consumption by Russian youth showed a pronounced lack of interest in the political agenda. The respondents living in three cities of Russia with over one million population helped to formulate questions related directly to political content, consumption platforms, and channels for receiving political news; we also attempted to assess the popularity of such content.

Students rated “politics” fifth among their thematic preferences, which meant it ranked below culture, fashion, social issues, and news about personal environment.

For schoolchildren (*Table 1*), information about the authorities and the state was the least interesting topic (21.7%). It is remarkable that the residents of Nizhny Novgorod (23.6%) were more politicized than the young Muscovites (22.9%). Schoolchildren in Rostov-on-Don were the least interested in politics (18.6%). It should be noted that information about human rights, and the ability to live, work, communicate, and express an opinion (27.5%) were more important and valued ty these schoolchildren than news about politics, especially among the schoolchildren in Moscow.

**Table 1:** Key motivation of media consumption of school children

	Total, number of people	Total, %	Moscow, number of people	Moscow, %	Rostovon- Don, number of people	Rostovon- Don, %	N. Novgorod, number of people	N. Novgorod, %
Interesting and beautiful (new films, cartoons, interesting facts about nature, animals, toys, cafes and restaurants, celebrity life, etc.)	1859	62.6	625	63.2	612	61.8	622	62.8
Business news, stories about large companies and money (loans, income and expenses, salaries, etc.)	582	19.6	238	24.0	142	14.4	202	20.4
Fashion and lifestyle (beautiful clothes, new looks, accessories, fashion trends, etc.)	1090	36.7	395	39.9	360	36.4	335	33.8
Useful content about casual life (how public transport runs — trolleybuses, buses, metro, where they will build new parks, roads, opened metro stations, etc.)	867	29.2	298	30.1	262	26.4	307	31.1
Important things about country and state (news about the president, about the prime minister, about the State Duma, various political actions, parties, etc.)	644	21.7	226	22.9	184	18.6	234	23.6
Ecology (environmental issues, water and air pollution, animal welfare, global environmental issues)	593	20.0	198	20.0	198	20.0	196	19.8
Human rights (ability to live, work, communicate and express his opinion, medical care, education etc.),	817	27.5	304	30.7	250	25.2	264	26.7
Local news (information about friends, family, relatives).	1538	51.8	510	51.5	538	54.4	489	49.4
No answer	90	3.0	0	0.0	41	4.1	50	5.0
Total	2970	100.0	990	100.0	990	100.0	990	100.0

Among the platforms where students consumed political content (information about the President, the Prime Minister, the State Duma, various political actions, parties, etc.), the leading position was taken by Internet sites and social networks. The reason for this is that these distribution channels are the most popular among youth, regardless of the content. However, it is worth noting that television has become an almost equally popular source of political information (*Table 2*).

**Table 2:** The main platforms for the political content of students

	Total, number of people	Total, %	Moscow, number of people	Moscow, %	Rostov- on-Don, number of people	Rostov- on-Don, %	N. No- vgorod, number of people	N. Novgorod, %
Internet sites	868	62.9	299	64.0	272	61.4	297	63.1
Social networks	775	56.1	279	59.7	242	54.6	254	53.9
Television	700	50.7	233	49.9	203	45.8	264	56.1
Messengers	235	17.0	110	23.6	66	14.9	59	12.5
Radio	83	6.0	20	4.3	24	5.4	39	8.3
Newspapers, magazines	97	7.0	26	5.6	30	6.8	41	8.7
Total	1381	100.0	467	100.0	443	100.0	471	100.0

Of course, the political situation is actively represented on television: daytime talk shows, analytical programs, opinion pieces, and news releases. But, is terrestrial TV really becoming a significant source of political information for digital youth and able to compete with online sources? Turning to the main topics that seemed interesting to those who responded about television (that is, at those moments when they really consumed TV content), politics turned out to be only in the fifth place, only ahead of civil law information, economic news, and the environmental agenda, which are definitely more complex and highly specialized topics.

It is also worth analyzing the key circumstances under which the digital generation watches television: these are indirectly related to the content of the shows. Importantly, TV consumption is first of all background viewing, fulfilling the desire to have fun and relax, or the traditional way of spending collective leisure time in Russian families; thus, there is a low level of involvement in what is happening on the screen (*Table 3*). The formula for interaction with political content on TV was very vividly described by one of the participants in his in-depth interview: “No, I never watch it at all. I don’t like politics, and if there is something important, my grandma will tell me; she will tell everything in brief.” The total amount of data we obtained indicates a low interest by the digital generation in politics and an unwillingness to receive political information in the process of television consumption.

**Table 3:** Key motivation of TV consumption of students

	Total, number of people	Total, %	Moscow, number of people	Moscow, %	Rostovon- Don, number of people	Rostovon- Don, %	N. Novgorod, number of people	N. Novgorod, %
To relax/ have fun/ if I’m bored	292	55.2	93	53.4	100	58.5	99	53.8
When I want to watch my favorite shows, movies, TV series on the big screen	252	47.6	87	50.0	82	48.0	83	45.1
I turn it on as a background and do my business in parallel	226	42.7	100	57.5	50	29.2	76	41.3
If I see that there is some interesting program going on / when I get carried away with an interesting program	216	40.8	75	43.1	67	39.2	74	40.2
I usually watch TV with my parents/siblings/ other relatives	124	23.4	49	28.2	30	17.5	45	24.5
I watch sports programs, matches, competitions, championships	119	22.5	26	14.9	60	35.1	33	17.9
To get information about my country, the world, politics	107	20.2	37	21.3	34	19.9	36	19.6
Total	529	100.0	174	100.0	171	100.0	184	100.0

On the other hand, the issue of young people’s political activity is increasingly appearing in public discussions, and the participation of their representatives in various events and rallies is being used by politicians to promote their own agenda both positively and negatively. What is happening today started in 2016, when some representatives of the younger generation suddenly turned from a politically passive group into an active one and became a stumbling block for the state. The government, having traditionally relied on federal television in the hope that it would be the only source of information and propaganda, overlooked the Internet and social networks – the main channels of information for generation Z, which the opposition forces quite successfully used.

The main protests of that year were attended mainly by young people ages 16 to 20, people who, as they said themselves, feared the future and the consequences of what is happening today. According to the calculations of the RANEPA School of Topical Humanitarian Studies, slightly more than 60% of respondents from the age group of 18-24 came to a rally for the first time in 2021. Thus, on the one hand, the interest of young people in politics cannot yet be called great, and on the other hand, their protest potential is quite large and periodically translates into action.

Experts distinguish several main reasons for this trend. Firstly, the absence of the so-called “Soviet mentality.” Young people are not afraid of being fired and losing benefits provided by the state. Secondly, mobilization through social networks plays an important role. According to media reports, the 2017 anti-corruption campaign united so many teenagers and high school students that many people associated this mobilization with an active information agenda in digital media. But the reason does not lie only in alternative channels of media consumption: young people understand that the formally democratic political system of Russia in reality differs from the social and historical values of democracy. Representatives of the digital generation interviewed by researchers say that the key values for them are morality, justice, and honesty (Bulletin, 2019).

The paradoxical nature of the youth interaction with political content is also reflected in our results: during the qualitative interviews, we noticed a pronounced desire for information and attention to the political situation among some respondents. The desire to get oriented in the surrounding reality, and to be aware of political news (“I want to know what is happening in the country, in the world...”, “I always want to be in the loop; it is important to know what everyone is discussing today, the last week, to understand what is happening and why”) can be found in this group. Moreover, the desire to know the latest news is also conditioned by the need for security (“What if there is a war? We need to prepare”). The desire to be aware of what is happening is explained by social needs, such as self-actualization, self-expression, the need to maintain conversation with peers, and to be interesting. (“At school, with friends, of course we discuss the news; well, how you can communicate without it? It’s necessary to somehow seem like adults”).

The need for generation Z members to discuss political issues is also connected with the fact that at this age, “they react more acutely to what is happening and need reinforcement of their point of view from surrounding adults or peers” (Russian Generation Z: Attitudes and Values, 2019-2020). Social networks generally allow young people to understand political and other problems: popular bloggers translate the language of TV channels (official news) into a simpler and more understandable one. This format is relevant to kids, teenagers, and freshmen.

At the same time, experts are paying attention not only to the children’s desire to understand the state of affairs, but also to their desire to change the world. According to the latest research conducted annually by the School of Public Policy of the Russian Presidential Academy of National Economy and Public Administration, teenagers put morality, justice, and honesty at the top of their concerns. One typical example was the statement that 20 years from now, I don’t want to be ashamed of what I did today. This concept of shame often raised its head in the interviews that this group of researchers worked on. They feel that they are the most active, relevant, and new generation with potential; however, this feeling is easy both to kindle and to suppress.

All of the above leads to an important idea: emotions play an essential role for Generation Z. New information and content providers (particularly on YouTube) are competitive with traditional media and resources not because of the information they offer, but due to the fact that their content is delivered with and arouses emotions. Emotion is the key to fame on the Internet, which is why the most popular bloggers and vloggers among young people are also young themselves; they share the same values, understand each other’s feelings, and broadcast everyday emotions. (Vyugina, 2019). “They are surrounded with gadgets, disconnected from offline communication and closed up in the virtual world - the only thing that shows that they are still alive socially is emotions,” summarized Evgeny Volkov, digital director of Life Media, during the Moscow Media Communication Forum in 2017.

Vkontakte analysts explain the success of young community administrators as follows: they operate with a huge audience, since they do not mention the news, its source, or essence, but speak mainly about things that arouse emotions in their readers: joy, sympathy, pride, or indignation. Their audience shares this outlook. The same happens with political news: it regularly appears in digital space and is actively consumed by youth on the Net, but its format differs from the usual political news that is broadcast, for example, on television. Therefore, young people’s ideas of the political situation are formed through the prism of their emotions.

Thus, on the one hand, political content has little interest for the generation in comparison with other topics; on the other hand, there is an obvious need to be aware of the current political situation, and new formats, primarily entertainment and gaming, stir young people’s various emotions, and contribute to their familiarity with it.

## Conclusion

The first stage of our study identified the importance of entertainment content as a key source of information for the digital generation, which gave us the opportunity to hypothesize about new formats of political news that are attractive to youth on the Net. In order to obtain in-depth data on the specifics of the consumption of political content, an additional qualitative study was conducted, in which respondents were offered six news topics related to the internal policy of the Russian Federation, presented in a visual humorous (memes and collages) or game format.

These in-depth interviews provided a clearer idea of where young people get political news, and how to resolve the contradictions in the media consumption of the younger generation.

The first and most important conclusion we drew is that the digital generation is familiar with the key political issues. Moreover, its members understand the humor embedded in a meme, or the irony on which a game project is based. Respondents described the emotions that arose after watching the news in these new formats with the words “funny, hilarious, laughable, cool.” We noticed that when describing their emotions from the perception of memes, young people tended to use expressive vocabulary, which also indicated a high degree of emotional involvement in interacting with the memes (Lysenko, 2017).

They also understood the entertaining and humorous nature of the proposed memes and games. In a meme, a comic effect arises from a combination of a funny picture and an equally funny signature; a meme is a metaphor, an allusion, a comparison which highlights the event that is perceived by a youth audience as absurd, irrational, meaningless, unfair, and even hopeless. Commenting on the memes, many respondents repeated the word “absurd” in relation to the meme’s subjects. The meme pushes the absurdity of situations to the limit by means of the satirical grotesque, which attracts young people. On the other hand, this exaggeration is directly related to frustration: laughter is the only active reaction (“it’s strange that we can’t do anything about it or change it”).

Respondents saw a tragic aspect in the mocked phenomena, along with the comic one. The sense of tragedy was based on bewilderment, indignation, and disappointment in everything connected with the Russian political agenda. Tragedy arises as a conflict between the ideal and the real. Internet memes, as well as political caricature, are characterized by “an air of dissatisfaction, discontent, disappointment in the surrounding political reality” (Kanashina, 2017).

The meaning embedded in memes was easily decoded. No difficulties were found in interpreting the plots; no false or inadequate perception of the offered content was encountered. The young people recognized and understood the metaphors and allusions, and easily decoded the language and visual allusions. Associations with events and decisions of politicians and the statements that were at the basis of the viral content instantly popped up in their minds.

Those memes that required deeper decoding turned out to be the most appealing. Their attractiveness and virality were associated with the satisfaction of overcoming difficulties in decoding, feelings of their own originality from the revelation of meanings, and feeling included in the “circle of initiates.”

Our study showed that young people actively use memes in their media practice not only for the emotional purpose of supply or relaxation. Another significant function of the meme for young people is a contact-establishing function. Memes are a marker, an identifier of “friend or foe” (Lysenko, 2017; Shomova, 2019), especially at this age when the main aim is the search for identity and relating oneself to a certain group.

New formats become news topics themselves. Journalists create materials in which they offer a selection of relevant memes on a topic for a good reason: this constitutes work with user content aimed at familiarizing a young audience with the political issues. However, not all representatives of youth on the Net are encouraged by a meme or a game to understand an issue more deeply and in more detail: they get a rather superficial knowledge, as well as a subjective, often critical view of the problem or story.

Our respondents noted that there is a ridiculing of the acutely topical political issues, bringing them to the point of absurdity by artistic means (satire, or grotesque political caricature). Our study revealed the lack of respect for authorities in the political space: if everyone can be laughed at, then no one is perfect and a role model. Thus, there is a desacralization of politicians and the public policies approved by the majority, which is comprised of older people. The comic interpretation of the previous idea of ethics in political activity, limits on dealing with personality issues in digital reality, the Russian attitude to tolerance, and other topical socially significant phenomena makes these digital formats extremely popular and viral among Russian youth.

Our study showed that respondents send or receive at least 5-6 memes from friends per day. The virality and the massive and lightning-fast distribution of memes are explained by such phenomena as the social exchange of emotions (Dunas & al., 2021). The discussion of the process of re-experiencing emotions during interpersonal interaction was introduced by psychologist Bernard Rimé (Rimé, Mesquita, Philippot, & Boca, 1991). In addition, Rimé identified the reasons that lead to people sharing their emotions (2007). The reasons our respondents shared memes partially reflected the reasons identified by Rimé: entertainment (a way to relax); attracting attention (the desire to impress friends); rapprochement (the desire to keep in touch with other people through the exchange of memes, avoid loneliness); fun (the desire to express emotions related to the topic of the meme); empathy (the desire to excite a friend); re-experiencing (the desire to remember or relive the event played out by the meme); and clarification (explaining important details of events through memes).

Of all the respondents, only one replied that he was ready to actively express his political position: to vote and go to rallies. The rest were critical and regretted that everything is not happening the way they would like, but were passive. Evaluating a meme or desire to share it was one of the few ways of showing one’s attitude toward a political phenomenon. The research showed, that, despite the low level of young people’s politicization, political processes caused an emotional response among young people. Our results correlate with the results of the All-Russian VCIOM survey “Youth and Politics: Current Challenges” (VCIOM, 2017). As in our study, the VCIOM survey showed that young people assess the situation inside the country as not meeting their core values. Almost half (45%) of 18-20 year-olds (VCIOM sociologists called them “generation zero”) pointed to violations of justice; 66% saw a lack of honesty; and 57%, a lack of respect. Our research showed that the value crisis is assessed by the young as a departure from the principles of modern civilized society.

Our audience also viewed political games with interest. The motivational basis of any game, according to Izard, is made up of emotions of joy. Winning a game is always a joy. Publishing news in a game format actually guarantees the attention of the young: the possibility of interactivity, the habit of playing various online games, and humor. The game adds the opportunity not only to laugh, but also to take some kind of action vis-a-vis a political person, to be an insider who knows the buzz words, to become a participant in politics, and sometimes to try out different scenarios for contributing to the political situation.

To summarize, we emphasize once again that the biggest emotional response among our respondents was caused by memes and games with a comic and satirical effect. Our respondents noted that they evoke such basic emotions as joy, surprise, amazement, and sometimes anger and disgust. (as defined by Ekman [2007])

Thanks to the entertainment formats, young people recharge their emotional energy. They help them cope with huge loads of work at the university and school that didn’t exist in their parents’ childhood. And laughter also helps them to endure the feelings of loneliness, dissatisfaction, and inferiority that arise while observing someone else’s successful life on social networks.

It is important to note that, as a format, such materials are single use in nature: interest in them most often disappears after the first failure or success. Moreover, the connection of the game project with the news topic is often very indirect, and the gameplay takes time. However, as a tool for obtaining informative content or the historical context of an event, games can become an easy way to enlighten a young audience.

## Discussion

A common misconception of researchers, representatives of the state system, and journalists is that the digital generation is not interested in politics. Sometimes this belief is supported by the passive civic behavior of members of that generation: refusal to participate in elections, backed up by the belief that one vote cannot change anything. The elderly average age of statesmen making decisions in the country today does not contribute to improving political communication. However, changes are gradually taking place. For some, political involvement becomes an attribute of their lifestyle (Gureeva, Dunas, & Muronets, 2020); for others, a struggle for social justice in the long term and a desire to influence the political system. However, familiarity with political content has acquired a broad reach due to the new formats of current news on digital platforms, which are the main source of information for Russian youth.

Emotions are the key to drawing the attention of a young audience. The popularity of entertainment content among young people has led to the emergence of new formats for presenting political news, which are actively resorted to, including popular media among the generation. Memes, games, and collages with humorous overtones regularly pop up in the news feeds of youth representatives and become a key source of political news.

The digital generation mainly consumes political news only through entertaining, often humorous, formats, so their idea of the situation in the world and in Russia is very subjective. At the same time, such media consumption is characterized by a rather superficial acquaintance with a problem, topic, or news.

The main motivation for the consumption of entertainment content by the digital generation is the youth’s need for positive emotions; thus they treat political news with humor, although this generation can critically assess them as well. Both the meme and the game certainly have a profound effect on the recipient due to being creolized texts. The texts containing visual components have a stronger impact on both the recipient’s consciousness and subconscious. For this reason, texts with visual elements (photos, videos) are actively used in political discourse and in political communication.

The authors believe that these features of media consumption should be taken into account by both media managers and political forces, since in the medium term, the digital generation will become the core of the electorate. Its representatives cannot be called extremely critical or objective; however, it is very important to choose the right form, format, and channels for distributing political news, as well as to analyze the emotions that are evoked in youth in the process of news consumption.

It was surprising for the Russian government that young people would participate actively in the political life of the country: before the demonstrations in 2019, they had not been taken into account at all. Since then, relations between the government and youth have been developed with great difficulty. The lack of interaction between the government and Generation Z as an important problem was expressed passionately by a famous Russian film director, Alexander Sokurov: “The state is making a big mistake, behaving so cavalierly with young people, with pupils and students. <...> You cannot start a civil war with schoolchildren and students. We must listen to them. But none of our politicians wants to listen to them, no one talks to them. <...> They are afraid of doing this. Why? It’s impossible, it’s impossible to tolerate it anymore” (*Kommersant*, 2016).

Some GenZ representatives escape the reach of propaganda, because their information channels do not match the main propaganda channels used by the government. Since we assume that the Generation Z in Russia is a generation of contradictions, the downside is its credulity for some other sources of political information, including some stories made up by politicians with alternative agendas. We cannot call Gen Zs highly critical and objective; the secret of success in communicating with them is not only the content itself, but also the form, the format, the channel you use to reach them as an audience, and the emotions you express.

For some reason, all the actions undertaken by the government still seem too artificial; they influence a very small proportion of the youth generation, and even the positive feedback they get might not be quite sincere. The results of the interaction are usually shown on TV to convince the older audience that the key to successful contact between the government and the young has been found. And that is why it is important for all state and local politicians, and first and foremost, the politicians who will replace the current ones, to find a way to reach the young audience in a more sustained way.
